# Sequencing methods and datasets to improve functional interpretation of *sleeping beauty* mutagenesis screens

**DOI:** 10.1186/1471-2164-15-1150

**Published:** 2014-12-19

**Authors:** Jesse D Riordan, Luke J Drury, Ryan P Smith, Benjamin T Brett, Laura M Rogers, Todd E Scheetz, Adam J Dupuy

**Affiliations:** Department of Anatomy and Cell Biology, University of Iowa, Iowa City, IA 52242 USA; Center for Bioinformatics and Computational Biology, University of Iowa, Iowa City, IA 52242 USA; Department of Ophthalmology and Visual Sciences; Roy J. & Lucille A. Carver College of Medicine, University of Iowa, Iowa City, IA 52242 USA

**Keywords:** Sleeping Beauty, Mutagenesis, Mouse models of cancer, Tumor clonality, Transposon remobilization, High-throughput sequencing

## Abstract

**Background:**

Animal models of cancer are useful to generate complementary datasets for comparison to human tumor data. Insertional mutagenesis screens, such as those utilizing the *Sleeping Beauty* (SB) transposon system, provide a model that recapitulates the spontaneous development and progression of human disease. This approach has been widely used to model a variety of cancers in mice. Comprehensive mutation profiles are generated for individual tumors through amplification of transposon insertion sites followed by high-throughput sequencing. Subsequent statistical analyses identify common insertion sites (CISs), which are predicted to be functionally involved in tumorigenesis. Current methods utilized for SB insertion site analysis have some significant limitations. For one, they do not account for transposon footprints – a class of mutation generated following transposon remobilization. Existing methods also discard quantitative sequence data due to uncertainty regarding the extent to which it accurately reflects mutation abundance within a heterogeneous tumor. Additionally, computational analyses generally assume that all potential insertion sites have an equal probability of being detected under non-selective conditions, an assumption without sufficient relevant data. The goal of our study was to address these potential confounding factors in order to enhance functional interpretation of insertion site data from tumors.

**Results:**

We describe here a novel method to detect footprints generated by transposon remobilization, which revealed minimal evidence of positive selection in tumors. We also present extensive characterization data demonstrating an ability to reproducibly assign semi-quantitative information to individual insertion sites within a tumor sample. Finally, we identify apparent biases for detection of inserted transposons in several genomic regions that may lead to the identification of false positive CISs.

**Conclusion:**

The information we provide can be used to refine analyses of data from insertional mutagenesis screens, improving functional interpretation of results and facilitating the identification of genes important in cancer development and progression.

**Electronic supplementary material:**

The online version of this article (doi:10.1186/1471-2164-15-1150) contains supplementary material, which is available to authorized users.

## Background

Animal models of cancer represent a complementary approach to direct analysis of patient tumors, allowing a level of experimental control not possible with human studies. The ability to generate large cohorts under precisely controlled conditions facilitates the interpretation of immensely complex datasets obtained from human samples. Currently, one of the greatest challenges to the development of successful cancer therapies is distinguishing so-called “driver” genetic aberrations that functionally contribute to tumors from background “passenger” events that are propagated during tumor development due to their co-occurrence with drivers. Insertional mutagenesis screens in animal models are particularly suited to addressing this issue, as they can provide large collections of tumors with tagged mutations that are considerably less complex at the molecular level than human cancer.

The *Sleeping Beauty* (SB) transposon system has proven useful for identifying drivers of tumorigenesis in a wide variety of tissue types [1], and it offers several advantages as a forward genetic screening tool. Mutagenic transposons have been engineered to be capable of inducing both gain- and loss-of-function mutations, allowing efficient identification of oncogenes and tumor suppressors, respectively. Insertion sites can easily be amplified following tumor development by taking advantage of unique sequence tags within each transposon, allowing the rapid generation of detailed mutation profiles. The ubiquity of the recognition site for transposon integration (a TA dinucleotide) provides the potential for an unbiased mutation pattern, allowing the identification of tumor-driving events throughout the entire genome. Another advantage of the SB system is its ability to closely recapitulate the process of tumorigenesis as it occurs in humans. Somatic mutations accumulate in a stepwise manner, driving a micro-evolutionary process within the developing tumor wherein those mutations that confer a selective advantage to cells are preferentially maintained. Positive selection for these mutation events leads to selective clonal expansion of the cells harboring them. Common insertion sites (CISs) are identified as regions of recurrent transposon insertion in multiple independent tumors, and they generally impact the function of a specific gene. Genes identified as CISs in this manner represent strong candidates whose mutation may serve as a driving event during cancer development.

As mentioned above, the ease of identifying mutations in SB-induced tumors through amplification of transposon/genome junctions is a major advantage of the system. There is, however, another class of mutation that can be generated through SB transposition that is not detected by current sequencing methods. Members of the *Tc1/mariner* family of DNA transposons, which includes SB, utilize a cut-and-paste mobilization mechanism that involves the generation of staggered double-strand breaks at the transposon inverted terminal repeats (ITRs). Following excision, three nucleotides derived from the transposon’s ITRs are left behind, generating a “footprint”. Because transposon integration involves duplication of the TA dinucleotide target site, transposon remobilization results in the insertion of five base pairs (bp) at the donor locus [2, 3]. Initial mobilization of a transposon from the donor concatemer to a distinct acceptor locus leaves a footprint between adjacent transposons at the donor site and is predicted to be functionally inconsequential. Remobilization of the inserted transposon from sites outside the donor concatemer within the same cell or its progeny, however, has the potential to significantly impact gene function. For example, a footprint caused by insertion within a coding exon and subsequent excision generates a frameshift mutation. To date, the prevalence of footprints in SB-induced tumors has not been assessed. Given the potential of these mutations to functionally contribute to tumorigenesis, it is important to characterize the rate at which they occur, as well as any evidence of their positive selection in SB-induced tumors. We describe here a high-throughput method to detect and validate footprints caused by transposon excision. Using this method we found that the rate of transposon remobilization in SB-induced tumors is relatively low and that the resulting footprints do not appear to be under strong positive selection. Our results suggest that this type of mutagenesis is unlikely to contribute significantly to tumor development in SB models of cancer.

High-throughput sequencing approaches produce millions of reads derived from transposon integration sites that map to tens of thousands of distinct genomic loci. Such complex patterns of insertion are likely the result of intratumoral heterogeneity and ongoing transposon mobilization caused by sustained transposase activity within the cells of a developing tumor mass. The inclusion or removal of background sites (*i.e.* insertion events present in a small minority of tumor cells) prior to downstream analysis will have profound effects on the quality of the resulting data set. Failure to remove background sites that are not subject to positive selection and clonal expansion during tumorigenesis decreases the sensitivity and accuracy of CIS identification [4]. While it is impossible to identify all of them, many background sites can be distinguished based on low abundance within a tumor or presence within a region of the genome subject to preferential detection as a result of biases in insertion pattern, amplification, sequencing, or mapping. With current sequencing methodologies these qualities are difficult to assess since the correlation between individual template abundance and sequencing depth has not been established, nor has the extent to which biological or technical biases influence the data been determined. Here we show that transposon insertion site data can be interpreted semi-quantitatively, permitting highly reproducible stratification based on relative abundance and associated clonality. We also report, based on extensive characterization of unselected insertions in normal tissues, apparent biases for detection of several specific genomic regions, an important factor to consider when inferring the degree of positive selection in tumors. Our findings provide novel insights into functionally important aspects of insertional mutagenesis screens with significant implications for interpretation of the data they generate. Incorporation of this information into tumor mutation profile analyses will enhance predictive ability, promoting effective discovery of strong candidate cancer genes for subsequent study.

## Results

### Characterization of transposon remobilization in tumors

We devised a sequencing strategy (Additional file 1: Figure S1) to detect footprints generated by transposon remobilization in a set of three T-cell acute lymphoblastic leukemias (T-ALLs) developed in triple-transgenic mice carrying alleles for 1) Cre recombinase expressed from the *CD4* promoter, 2) Cre-inducible SB transposase, and 3) a concatemer of T2/Onc2 mutagenic transposons [5]. The TG6113 T2/Onc2 strain was chosen because it has the highest copy number of transposons within the donor concatemer of any existing SB strain [6], and the *CD4*-Cre model was chosen due to its long latency relative to other T2/Onc2-induced malignancies [5]. These characteristics of the selected model impart the highest predicted rate of transposon remobilization possible in SB-induced tumors, providing the greatest sensitivity to detect footprints.

Genomic DNA extracted from the tumor samples was sheared to an average size of 300 bp, followed by end-repair and ligation of a blunt-ended adaptor sequence. Next, samples were digested with *Hpy*CH4III, a restriction endonuclease that recognizes the five bp sequence left behind following SB transposon excision, and a second adaptor sequence was ligated to the 3’ overhang left by *Hpy*CH4III. PCR was performed with primers designed to amplify fragments containing both adaptor sequences, and the resultant products were sequenced on the Illumina platform. A total of 95,294,646 sequence reads were mapped amongst the set of three samples.

With four defined base pairs, the *Hpy*CH4III recognition sequence (5’-ACNGT-3’) is present in the mouse genome at over seven million sites. We designed the high-throughput footprint detection method to enrich for footprints using primers specific to the two consensus footprint sequences [5’-TACAGTA-3’ and 5’-TACTGTA-3’] (Additional file 1: Figure S1). While these sequences are far less frequent than *Hpy*CH4III sites, they are still present at over 300,000 locations in the mouse genome. Therefore, a tissue-matched normal sample lacking SB transposition was processed alongside the tumors in order to distinguish actual footprints from endogenous sites with the same sequence. Any sites detected in the normal sample were removed from the tumor-derived sequences prior to further analysis. Another source of background in this experiment comes from transposon insertions that have not been remobilized; every junction between an SB transposon ITR element and the genome contains a recognition site for *Hpy*CH4III. To eliminate this background, any sites that were identified as harboring clonally expanded transposon insertions were also removed prior to further analysis.

**Figure 1:**
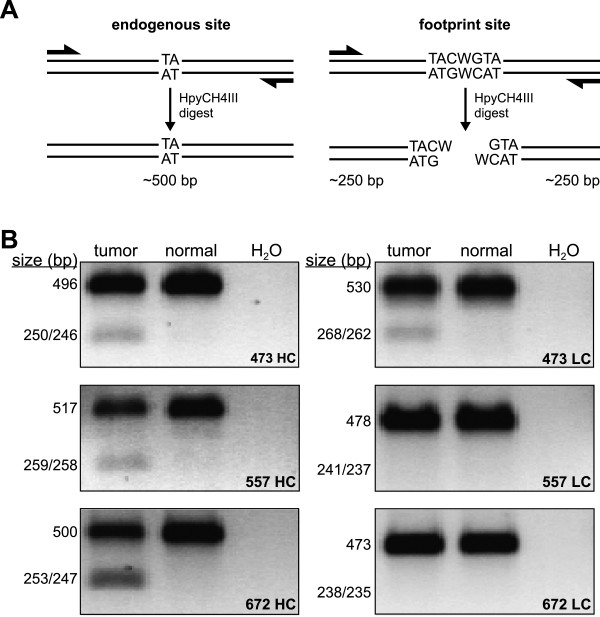
**Validation of candidate SB-induced footprint sites identified by deep-sequencing analysis. (A)** Schematic diagram depicting the method used to validate putative footprints. Primers were designed to amplify genomic regions predicted to contain SB-induced footprints in DNA from tumor samples, but not in DNA from normal tissue. Products amplified from tumor and normal DNA were purified and subjected to enzymatic digest with *Hpy*CH4III, a restriction endonuclease whose recognition site is generated following SB transposon excision. **(B)** Digested products were separated by agarose gel electrophoresis. Results from a high confidence (HC) and low confidence (LC) predicted footprint are shown for each of the three tumors analyzed. Undigested products range in size from 473–530 bp (upper bands), while digested products range in size from 235–268 bp (lower bands).

This approach provided a list of putative transposon footprint sites for each tumor sample (Additional file 2: Table S1). Candidate footprint sites were designated as high or low confidence based on a comparison of their sequencing depth to that of simultaneously sequenced loci that had previously been identified as clonally expanded transposon insertion sites. To verify the presence of a footprint, PCR was performed to amplify 10 candidate loci from each tumor. Products were digested with *Hpy*CH4III and separated by agarose gel electrophoresis (Figure 1). Using this strategy, we confirmed the presence of transposon footprints in 100% (12/12) of analyzed high confidence sites and 5.6% (1/18) of analyzed low confidence sites (Table 1), leading to estimates of 153, 139, and 8 actual footprints in the set of three tumor samples. Candidates were further categorized based on localization within regions encoding genes. Overall, 38.6% and 2.4% of sites fell within introns and exons, respectively. Roughly 34.1% of mapped TA dinucleotides in the current mouse reference genome (GRCm38/mm10) are located within introns, while ~1.5% are located within exons. If footprints functionally contribute to tumorigenesis, we would expect to detect them within exons (due to the nature of the induced mutation) more frequently than predicted by chance as a result of positive selection pressure. Our results demonstrate a lack of significant difference between the expected and observed proportion of footprints mapping to exonic TA sites (p = 0.076, two-tailed two-proportion z-test), providing minimal evidence of such enrichment. Nevertheless, our results do demonstrate that SB-induced tumors will harbor rare frameshift mutations caused by transposon footprints.

**Table 1:** **Candidate footprints identified in SB-induced tumors**

Tumor ID	Predicted high conf.	Validated high conf.	Predicted low conf.	Validated low conf.	Total candidates	Within introns	Within exons
473	149	5/5 (100%)	69	0/5 (0%)	218	82 (37.6%)	5 (2.3%)
557	2	2/2 (100%)	95	1/8 (12.5%)	97	41 (42.3%)	3 (3.1%)
672	133	5/5 (100%)	104	0/5 (0%)	237	90 (38.0%)	5 (2.1%)
**Total**	**284**	**12/12 (100%)**	**268**	**1/18 (5.6%)**	**552**	**213 (38.6%)**	**13 (2.4%)**

### Semi-quantitative stratification of insertion sites based on relative clonality

Typical high-throughput sequencing analyses of genomic DNA from tumors induced by transposon-mediated mutagenesis detect thousands of unique insertion sites in each tumor [4, 7]. The dynamic range of sequencing depth at each site can be quite large, often extending from a single read to tens of thousands of reads. In general, it is assumed that the extent of sequence coverage for a specific insertion site is a reflection of its prevalence within the tumor mass (*i.e.* the percentage of cells within the tumor harboring a transposon integrated at a specific genomic coordinate). Additional factors, however, such as PCR amplification bias and differential mapping efficiency could potentially impact coverage, complicating quantitative interpretation of insertion site sequencing data. Assessment of the influence such factors have on sequence coverage is therefore critical to enhancing the ability to infer relative clonality information for individual mutations within a tumor.

We conducted a standard curve experiment to determine the correlation between sequencing depth of a particular transposon insertion site within a tumor and its actual abundance relative to other sites. A set of 10 plasmids was engineered, each of which contained two artificial templates for amplification by ligation-mediated (LM)-PCR that consisted of either the left or right SB transposon ITR element adjacent to a specific genomic locus identified through prior mutagenesis screening (Additional file 1: Figure S2). For each construct, the paired templates were predicted to be subject to differential amplification bias (*e.g.* based on GC content or repetitive nature; see details in Additional file 3: Table S2). This physical linkage of templates with hypothesized amplification biases was included to allow a direct assessment of the magnitude of such biases, given the known shared abundance of paired templates. Standard plasmid DNA was mixed with tumor DNA such that each construct was represented at an abundance of 1.0, 0.5, or 0.125 copies per genome to mimic mutations present in 100%, 50% or 12.5% of tumor cells, respectively. We chose previously analyzed tumors from both high-copy T2/Onc2 [5] and low-copy T2/Onc3 [8] transposon-based screens to allow assessment of the influence that standard inclusion has on the detection of actual insertion sites and of the technique’s robustness across multiple sample types. An Illumina sequencing library was prepared for mixed DNA samples in triplicate using a shearing-based LM-PCR strategy [8], and a total of 11,650,096 reads were mapped amongst the eight samples.

**Figure 2:**
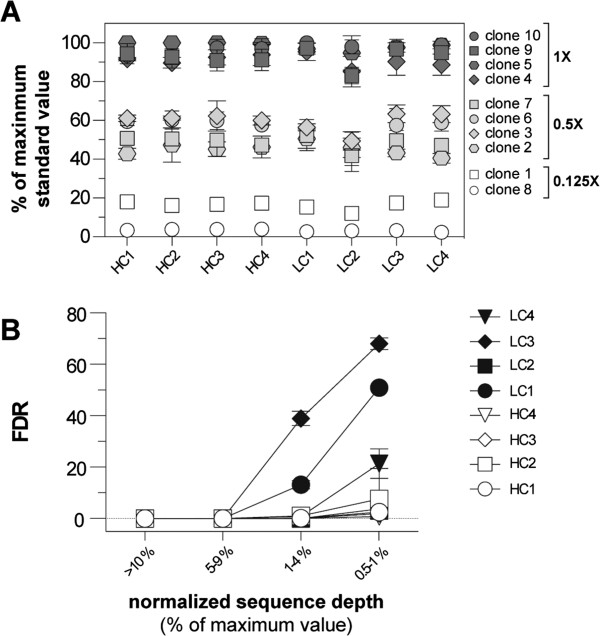
**The use of standards demonstrates the semi-quantitative range of ligation-mediated PCR to detect transposon insertions in a complex sample. (A)** A set of 10 plasmid standards were added to eight DNA samples from SB-induced tumors. These tumors were generated using either a high-copy (HC1-4) [5] or low-copy (LC1-4) transposon donor [8]. The standards were split into three groups and spiked into each tumor DNA sample to mimic insertion events present in 1 copy per cell (1X), 0.5 copies per cell (0.5X), or 0.125 copies per cell (0.125X). The maximum read value obtained for each standard was then expressed as a percentage of the most abundant standard identified in each sample. The indicated values represent the average value for each standard obtained from three independent sequence runs for each sample. **(B)** Insertion sites in each sample were grouped according to the normalized read value (% of maximum signal). The false discovery rate was estimated for each group of insertions (see Methods).

Following mapping, the number of sequence reads assigned to each transposon insertion site was normalized to reflect its proportion relative to the most abundant site (*i.e.* the top site was set to 100%). As shown in Figure 2A, sites derived from the plasmid standards reproducibly clustered into non-overlapping groups of those included at 100%, 50%, or 12.5% abundance. Interestingly, sequencing depth did not differ significantly between paired standard amplification templates. This finding indicates that fragments containing DNA elements predicted to confer PCR amplification bias, such as GC-rich stretches and repetitive regions, are amplified to a similar extent as fragments lacking these elements by the LM-PCR protocol utilized. Thus, read number can reliably be assumed to approximate relative template abundance. It should also be noted that the percentage scores assigned to genomic insertion sites were not significantly affected by inclusion of the standards (Additional file 1: Figure S3A). Based on analysis of each tumor sample in triplicate, a false discovery rate (FDR) for insertion site detection was calculated (see Methods). For those sites with a normalized abundance of 5% or greater, the FDR was zero (Figure 2B). Below 5%, the FDR increased modestly for tumors with high-copy transposon concatemers, with more significant increases observed for tumors having low-copy concatemers.

In a study of *piggyBac* transposon integration in cultured cells, Koudijs and colleagues found that the number of unique ligation points detected for each insertion site following shearing-based LM-PCR more accurately reflected clonality than sequence coverage [9]. To determine if this was also true for SB-induced tumor samples, we repeated our percentile rank analyses using the number of unique ligation points for each insertion site instead of read number. Although the rank order of sites derived from the plasmid standards was reproducible and approximated the expected result, separation between groups was greatly decreased as compared to the analysis based on read number, and distinction of groups was impossible due to overlap (Additional file 1: Figure S3B). The divergent outcome of our studies is likely explained by differences in the source of transposon templates and depth of sequencing. Importantly, our analysis was conducted in the context of complex and heterogeneous tumor DNA with extensive sequencing depth, conditions designed to maximize relevance to datasets generated by SB cancer screens.

### Assessment of detection bias in SB insertion site datasets

Several studies have analyzed genome-wide SB transposon insertion sites in an effort to identify any biases in the integration pattern. Aside from a local hopping phenomenon, in which transposon re-integration following excision has a tendency to occur within a ~5-10 megabase window surrounding the donor site [10], minimal insertion site bias has been reported for SB [8, 11–15]. The majority of these studies, however, have been conducted using cell culture systems with transposon delivery from transfected plasmid DNA and/or with selection for cells that have integrated transposons. We sought to generate comprehensive SB insertion site profiles from tissues *in vivo* in the absence of selective pressure in order to produce an unbiased dataset more closely matched to those obtained from SB-induced tumors.

To generate such a dataset, transposition was activated ubiquitously in eight-week-old mice and allowed to persist for two weeks prior to tissue collection. Inducible, ubiquitous activation was achieved through tamoxifen injection into offspring resulting from a cross between the homozygous *ROSA26-CreERT2* strain [16] and an SB strain homozygous for a Cre-inducible *ROSA26-SBase* allele and a concatemer of either T2/Onc2 (TG6070 and TG6113) or T2/Onc3 (TG12740 and TG12775) transposons. Transposon integration sites were identified in genomic DNA from liver, spleen, skin, and lung tissue by LM-PCR followed by Illumina sequencing. Three independent sections per tissue were analyzed for two males and two females of each strain (192 samples in all), and a total of 33,350,101 sequence reads were mapped to 599,938 unique genomic loci. In order to estimate the relative degree of clonal expansion occurring in tissues during the two week transposition period, we compared the percentage of all mapped sequence reads assigned to the top five most frequently identified sites between a set of T2/Onc3 transposon-induced liver tumors [8] and the T2/Onc3 normal liver samples. For tumors, an average of 19.4% (SD = 12.4%) of sequenced reads mapped to the top five insertion sites. In contrast, the average value was only 4.6% (SD = 4.1%) for normal liver samples. This difference is indicative of a lower level of clonal expansion in the livers subjected to two weeks of transposition, as compared to SB-induced liver tumors (p = 5.0E-7, Student’s t-test). Further evidence that minimal clonal expansion occurred in the normal samples is provided by the finding that 32.4% of insertion sites fall within genes, which is lower than the 35.6% predicted for random integration given the distribution of TA sites throughout the genome. These data indicate a lack of significant positive selective pressure imposed upon tissues during the two weeks of transposition.

We used a previously described computational strategy [17] to identify genomic regions with a higher number of detected transposon insertion sites than predicted from a random pattern of integration (see Methods). It should be noted that sequencing depth was not considered in this analysis; instead, the number of unique insertion sites within a region was used for calculations. Additionally, insertion sites from all 192 samples were pooled for the analysis and processed collectively. This was done to maximize statistical power after it was determined that, aside from local hopping, no major differences existed in the distribution of mapped insertion sites among independent samples, indicating high reproducibility of general transposition activity regardless of tissue type, gender, or strain. The mouse genome was divided into non-overlapping 20 kilobase (kb) windows and an algorithm was applied to compare the number of observed insertion sites in each window to the expected number, accounting for the prevalence of potential insertion sites (*i.e.* TA dinucleotides). After Bonferroni correction for multiple hypothesis testing, this analysis identified 794 distinct windows with significantly more insertion sites than predicted (p < 1.0E-7; Additional file 4: Table S3).

**Figure 3:**
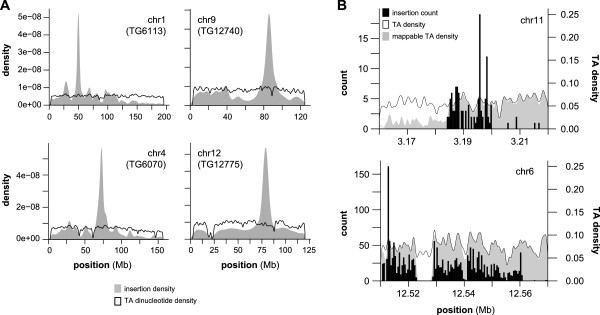
**Transposon insertion densities on local chromosomes and non-local windows. (A)** The transposon insertion site and TA dinucleotide densities are shown for each chromosome that undergoes local hopping. In each case, the height of the peak indicates the proportion of total events present at that location. The strongest single peak on each chromosome corresponds to the mapped location of the indicated transgene (*e.g.* TG6113). **(B)** Two genomic regions identified by PRIM analysis show enrichment for transposon insertion: chr11:3,180,001-3,200,000 (top) and chr6:12,510,001-12,570,001 (bottom). In each case, a histogram plot indicates the number of insertion events present at each location (left axis). The density plot indicated by the solid line shows the proportion of total TA dinucleotides (right axis). The density plot represented by the shaded curve represents an estimate indicating the proportion of TA sites in which more than 50% of read lengths can be accurately mapped (see Methods).

To improve our ability to evaluate the local hopping phenomenon, we first precisely mapped transgene insertion sites for the transposon concatemer in each of the four SB strains utilized. This was achieved through the design of PCR primer pairs that amplify the transposon/genome junction for each concatemer. PCR genotyping protocols for each transposon allele to distinguish wild-type, heterozygous, and homozygous mice can be found in the Additional file 5: Supplemental Methods section; typical results are shown in Additional file 1: Figure S4, along with the chromosomal coordinates of each transposon concatemer. As expected based on the local hopping activity of SB, the majority of significant windows (85.4%) mapped to one of the four local chromosomes. Of these windows, 86.1% are within six megabases of the concatemer, demonstrating that the majority of local hopping activity occurs within a fairly limited interval rather than being spread throughout the entire local chromosome. This point is illustrated graphically by Figure 3A, which shows the distribution of insertion sites detected in unselected tissues relative to that of all potential insertion sites for the local chromosome of each strain. After removing those on local chromosomes, 116 significant windows remain (Additional file 4: Table S3). These genomic loci define a set of non-local zones of preferential detection in tissues subjected to SB transposition without selection. As such, they represent potential regions of false-positive CIS identification in SB cancer screens.

One possible explanation for enhanced detection of transposon insertions within a specific genomic window is the improper mapping of sequence reads that lack a sufficient number of unique bases to be definitively assigned to a single locus. We identified several insertion sites in our dataset that are likely the result of mapping errors and confirmed the absence of integrated transposons at two of them (Additional file 1: Figure S5). Closer examination of the IAS pipeline revealed that the Bowtie2 alignment tool was reporting alignments to repetitive regions that could not be uniquely mapped. Not surprisingly, most (104/116; 89.7%) of the significant windows contained a tight cluster of insertion events that map to one or more TA sites within a repetitive sequence based on RepeatMasker annotation.

To eliminate this source of bias from our analyses, we developed an algorithm to determine the number of bases required for absolute mapping confidence at each TA dinucleotide in the genome. Using this information, we repeated our analysis to identify windows of preferential transposon detection after eliminating sequence reads that could not be mapped unambiguously. This approach identified only 16 non-local windows that had a significantly higher number of detected insertions than expected (Table 2), 12 of which were identified by our initial analysis. Density plots for potential and detected insertion sites within two of these windows are depicted in Figure 3B. Based on the amount of discrepancy between our analyses before and after removing ambiguous sequence reads, mapping artifacts appear to be a major source of potential bias for insertional mutagenesis screens. Consistent with our initial observation of similar transposon distribution across sample types, insertions were identified in all four tissues for 14 of 16 windows, with the remaining two windows containing insertions in three of four tissues. Similarly, insertions from all four or three of four mouse strains were identified in 9/16 and 7/16 windows, respectively (Table 2).

**Table 2:** **Map-corrected, non-local genomic windows with significant enrichment for detected transposon insertions in unselected tissues (liver, lung, skin, spleen)**

Window coordinates	p-value	Identified by initial analysis?	# of tissues with insertions	# of strains with insertions	Gene within window	Other notes
chr2:81,670,001-81,690,000	3.39E-07	No	3	3		
chr5:28,160,001-28,180,000	<1.00E-300	Yes	4	4	*En2*	Transposon artifact^1^
chr5:113,680,001-113,700,000	7.91E-18	Yes	4	3	*1700069L16Rik*	
chr6:12,510,001-12,530,000	<1.00E-300	Yes	4	4	*Thsd7a*	
chr6:12,530,001-12,550,000	<1.00E-300	Yes	4	4	*Thsd7a*	
chr6:12,550,001-12,570,000	<1.00E-300	Yes	4	3	*Thsd7a*	
chr8:65,840,001-65,860,000	7.21E-11	Yes	4	3	*March1*	
chr10:36,800,001-36,820,000	3.34E-15	Yes	4	4	*Hs3st5*	
chr11:3,180,001-3,200,000	5.64E-176	Yes	4	4	*Sfi1*	Genome assembly artifact^2^
chr11:76,780,001-76,800,000	3.30E-45	Yes	4	4	*Cpd*	
chr11:79,420,001-79,440,000	1.88E-09	Yes	4	3	*Nf1*	
chr13:15,380,001-15,400,000	1.95E-07	No	4	4		
chr13:31,620,001-31,640,000	1.01E-200	Yes	4	4	*Foxf2*	Transposon artifact^1^
chr16:28,880,001-28,900,000	5.71E-09	Yes	4	3	*Mb21d2*	
chr17:83,160,001-83,180,000	2.93E-08	No	4	3		
chrX:150,970,001-150,990,000	2.26E-07	No	3	4	*Gnl3l*	

^1^Detected as significant due to the inclusion of *En2* and *Foxf2* splicing elements within the transposon structure.

^2^Detected as significant due to presence at a higher copy number than annotated in the reference genome.

While further characterization will be required to conclusively determine the mechanisms responsible in each case, our results demonstrate that in the absence of selective pressure certain regions of the genome are subject to preferential detection by standard analytical techniques used to profile SB insertion sites. This is an important point to consider when assessing the distribution of transposon insertions in tumors; failure to do so could lead to overestimation of significance and increase the occurrence of false-positive CIS identification.

## Discussion

The relative simplicity of genetic profiling in animal models of cancer induced by insertional mutagenesis is a major advantage when attempting to elucidate molecular mechanisms of carcinogenesis. Because the insertional mutagen’s sequence is known, techniques that amplify adjacent genomic regions for sequencing can readily be implemented. It is important to note, however, that additional types of genetic aberrations are certainly present at some level in tumors induced by this mechanism. Of particular interest for cancer models induced by *Sleeping Beauty* transposition is the prevalence of mutations generated by transposons that remobilize following genomic integration. Compared to an untargeted locus, one that has been subject to transposon integration and re-excision is left with an insertion of five bases (the footprint). While this type of mutation could have functional effects regardless of where it occurs in the genome, the greatest impact would be expected within coding exons, due to the generation of frameshift mutations. Measuring the degree of transposon remobilization and selection for maintenance of footprints in tumors is important to ensure that functional contributions from these events are not missed when interpreting data from SB insertional mutagenesis screens. To date, no such characterization has been reported.

We have described a novel high-throughput sequencing approach to identify SB transposition footprints. Using this technique, we detected ~100-200 candidate footprints per sample in a set of three SB-induced T-cell leukemias (Table 1). Around half of candidate sites were predicted with high confidence based on sequencing depth. Given that only a small percentage of low confidence candidate sites could be validated, the actual number of footprints in each sample is likely to be smaller than the list of putative sites. The model that we analyzed was chosen based on its predicted high potential for transposon remobilization, so the phenomenon is expected to be even less frequent in other SB models. Further characterization of distinct SB strains and tumor types will be required to determine whether or not this is the case.

When considering the entire set of candidate footprints identified in the three samples we analyzed, frameshift mutations would result in two to three candidates per sample (Additional file 2: Table S1). This finding is consistent with the expected rate of one to four per sample based on the percentage of the genome that falls within an open reading frame (~1.5%). Similarly, the proportion of candidate footprints residing within exons did not differ significantly from expectation. Although analysis of a larger number of samples will be required to make definitive conclusions, our results indicate a lack of strong positive selection for transposon remobilization events in tumors, suggesting that they are unlikely to be a significant source of driver mutations in SB cancer models. SB-induced tumors have also been found to display minimal genomic instability [18]. Data such as these serve to increase confidence that detailed profiles of transposon integration sites are sufficient to provide a comprehensive list of likely tumor driving events in SB-induced models of cancer.

The relative clonality of specific mutations within a heterogeneous tumor mass is a metric with important implications. This information can provide insight into the timing, function, transforming activity, and cooperative potential of a given mutation or set of mutations, among other qualities. Particularly in the case of tumor models driven by insertional mutagenesis, where a large number of samples can be generated under controlled conditions, the ability to identify recurrent patterns of clonality for specific mutations is highly desirable and informative. It is, however, quite challenging to assign reliable quantitative information to individual mutations identified in a tumor mass. This is largely due to difficulties in correlating sequencing depth for a given mutation with its actual abundance, a problem that stems from experimental limitations including amplification bias and differential mapping efficiencies of disparate genomic loci. As a result, current methods of interpreting insertion data from mutagenesis screens do not consider the abundance of each individual site. Instead, some method of filtration is typically applied to remove background data, and all sites that pass the filter are treated equally in downstream analyses [8, 19–22]. This approach, although appropriate based on available data regarding the relationship between sequencing depth and mutation clonality, discards a wealth of information. A method allowing this information to be recovered and accurately interpreted would reveal patterns and relationships between mutations not previously appreciated, undoubtedly leading to an improved understanding of the molecular mechanisms driving SB-induced tumor formation.

Recently, strategies to decrease size-based amplification bias and increase sequencing depth have led to improvements in the quantitative interpretation of data from insertional mutagenesis screens [7, 9]; however, these interpretations are still quite broad and have so far been used only to trim CIS lists with the goal of eliminating background signal. We utilized the advancements presented in these studies (shearing of genomic DNA to minimize size-based bias during amplification and use of the Illumina sequencing platform to obtain in-depth tumor profiles) to conduct a rigorous assessment of the relationship between template abundance and sequencing depth. Our goal was to gain the ability to confidently assign quantitative significance to individual insertion sites based on sequencing depth. By mixing a series of standard templates for LM-PCR amplification with tumor DNA in a controlled manner, we were able to mimic mutations present in defined percentages of tumor cells. This allowed us to assess the accuracy and reproducibility with which each template could be detected by our sequencing method. When all insertion sites were ordered according to sequencing depth, standard templates included to mimic mutations present in 100%, 50%, or 12.5% of cells reproducibly clustered into three non-overlapping groups across three independent technical replicates. Additionally, the normalized percentage values assigned to each standard were within ~10% of the expected value. These results demonstrate that relative sequencing depth of specific transposon insertion sites within a tumor can be confidently interpreted as an approximation of their abundance using our LM-PCR method. This information can be used to infer the relative clonality of each mutation. It is important to note, however, that the results cannot be interpreted in a precisely quantitative manner to arrange individual mutations according to absolute abundance. Rather, mutations can be divided semi-quantitatively into distinct groups based on relative abundance. The correlation between sequencing depth and template abundance is insufficient to precisely arrange mutations within these groups.

We have demonstrated that sequencing depth can be interpreted semi-quantitatively to organize transposon insertion sites within a tumor into three major categories based on relative clonality. This information can be used to infer the relative timing of mutation acquisition during tumor development as well as to assess tumor heterogeneity. For example, a CIS that is recurrently detected as one of the most abundant sites in independent tumors is likely to be involved in the early stages of tumor initiation. Its pattern of mutation would suggest that the majority of cells within multiple tumor masses were derived from a cell harboring a transposon insertion within that region, indicative of strong positive selection during early tumor development, as well as selection for maintenance during tumor progression. On the other hand, a CIS that is recurrently detected at a relatively low abundance is more likely to be involved in later stages of tumor development. Detection of its repeated mutation in independent tumors would indicate positive selection for cells that acquire the mutation, but its presence at levels significantly lower than the most abundant mutations within a tumor would suggest acquisition at a stage of tumor development after significant clonal expansion had already occurred. The ability to classify CISs according to relative clonality within a tumor mass will significantly improve our understanding of the dynamic evolution of tumors induced by insertional mutagenesis, and it will allow mechanistic connections to be made between independent mutation events that would otherwise be impossible.

Current methods to analyze insertion site data from SB mutagenesis screens typically assume that all TA dinucleotides in the genome outside of the local chromosome have an equal chance of being detected as insertion sites in the absence of selective pressure. This assumption is based on studies that have characterized global patterns of SB transposition and found no major biases [8, 11–15]. One potential issue with applying the data from these analyses to data from tumorigenesis screens is that the experimental context is quite different. Whereas characterization of integration patterns has largely been conducted in cultured cells, often with transposons delivered from transfected plasmids, transposition happens *in vivo* from resident transposon concatemers in mutagenesis screens. Characterization studies also often apply selection for cells that have integrations, a process that could influence the pattern of detected sites. Recently, an in-depth analysis of unselected SB insertion sites in mouse embryonic stem (mES) cells reported some influence of transcriptional activity, regulatory function, and chromatin topology on target site selection [23]. There is remarkably little overlap between this dataset and ours – only two of the genomic windows we report as having detection bias show evidence of enhanced detection in the mES cell dataset. This result indicates substantial differences in SB transposition activity based on the experimental conditions utilized. Importantly, our approach characterized genome-wide insertion patterns from transgenic concatemers *in vivo* and in the absence of selection, conditions chosen to match techniques used for tumorigenesis screens. In this experimental context, patterns of detection bias do not appear to be influenced by tissue type or concatemer location, as evidenced by the presence of insertions from nearly all strains and tissues in all 16 non-local windows of preferential detection that could not be explained by mapping artifacts. Additionally, our study is the most comprehensive to date, analyzing five times more sites than the most extensive study conducted previously [23]. These qualities enhance the relevance of our data to insertion profiles from SB-induced tumors.

An alternative method to generate SB transposon insertion data from unselected tissue *in vivo* has previously been utilized. For this approach, integration sites are profiled in DNA from tail clips of weanling mice [24–26] or histologically normal tissue [27], and any site identified as a CIS in the unselected sample set is removed from the CIS list generated for tumors. Thus far, all such analyses have been performed using pyrosequencing, which has been shown to provide insufficient data for comprehensive CIS detection [4]. By using the Illumina sequencing platform, we were able to obtain profiles from unselected tissues at much greater depth, allowing us to conduct the most thorough analysis of background insertion profiles to date. Two of the regions identified by our method (chr2:98,650,001-98,670,000 and chr16:15,830,001-15,850,000) have previously been reported as background loci detected in tail DNA from SB mice. Our identification of another region (chr11:3,180,001-3,200,000) can be explained by overlap with a locus previously recognized to be present at higher copy than indicated by the mouse reference genome [28].

Consistent with our assumption of a lack of significant clonal expansion of cell subpopulations over a two-week transposition period, the transposon integration profiles for all of the normal tissue samples were strikingly different from those typically obtained from tumor samples. In tumors, the majority of mapped sequence reads are derived from a relatively small number of insertions that have increased in prevalence relative to other insertions due to clonal expansion of the cells harboring them, and many of these clonally-expanded sites are recurrently identified in multiple independent tumors. In contrast, sequence reads derived from insertion sites identified in normal tissues following two weeks of transposition were more evenly distributed, indicating a lack of significant selection for cells harboring any given insertion. Additionally, we did not observe enrichment for insertions within transcription units in normal samples, whereas tumors display such enrichment due to the clonal expansion of cells harboring driver mutations.

In total, our initial analysis identified 116 non-local genomic regions detected to have a significantly higher frequency of insertion than predicted based on a random transposition pattern. In some cases this may result from an increased prevalence for SB transposons to integrate within these regions relative to others (*i.e.* it may have a biological basis). Alternatively, it could be caused by technical biases in amplification, sequencing, or mapping. Any of these factors could contribute to enhanced detection of specific insertion sites, and it is likely that each of them does to some extent. In our dataset, mapping artifacts were responsible for the majority of preferentially detected windows. Removal of sequence reads that could not be definitively mapped prior to downstream analysis eliminated the majority of initially identified significant windows, leaving only 16 that were not attributed to this source of bias.

Regardless of the explanation behind the preferential detection of unselected transposon insertions within specific genomic regions, it is an important factor that should be considered when interpreting CIS lists from SB screens. The enhanced baseline detection rate for insertions within these regions should be accounted for to avoid overestimating the significance of their repeated identification in tumors. The importance of this point is demonstrated by the discovery that ~49% (29/59) of genes overlapped by the regions we identified have previously been reported as CISs in SB screens (Table 3), according to the Candidate Cancer Gene Database [29]. A gene’s presence on this list does not necessarily implicate it as a false-positive CIS. Instead, it indicates that the expected frequency of insertion within the locus should be adjusted to reflect its observed heightened rate of baseline detection. As such, genes within regions of positive detection bias must meet stricter requirements to confidently be called a CIS than they would under the assumption of completely random transposon distribution.

**Table 3:** **Potential false-positive common insertion sites reported in prior publications**

Gene symbol	Window coordinates	# of studies	References
Sfi1	chr11:3,180,001-3,200,000	12	[9, 21, 25, 26, 30–37]
Nf1	chr11:79,420,001-79,440,000	7	[7, 9, 27, 35, 36, 38, 39]
Abi1	chr2:22,990,001-23,010,000	5	[20–22, 27, 39]
Son	chr16:91,640,001-91,660,000	4	[21, 22, 27, 39]
Thsd7a	chr6:12,510,001-12,530,000; chr6:12,530,001-12,550,000; chr6:12,550,001-12,570,000	4	[5, 20, 39, 40]
Chl1	chr6:103,630,001-103,650,000	3	[27, 37, 40]
Erc1	chr6:119,570,001-119,590,000	3	[21, 22, 39]
Fbxl17	chr17:63,460,001-63,480,000	3	[20, 27, 39]
Lphn3	chr5:81,340,001-81,360,000	3	[22, 27, 39]
March1	chr8:65,840,001-65,860,000	3	[19, 31, 39]
Ahcy	chr2:155,050,001-155,070,000	2	[21, 39]
Diap2	chrX:130,130,001-130,150,000	2	[22, 39]
Faf2	chr13:54,620,001-54,640,000	2	[22, 39]
Fhit	chr14:9,840,001-9,860,000	2	[6, 27]
Gart	chr16:91,640,001-91,660,000	2	[21, 39]
Nav2	chr7:49,260,001-49,280,000	2	[7, 39]
Phf21a	chr2:92,270,001-92,290,000	2	[22, 39]
2610307P16Rik	chr13:28,800,001-28,820,000	1	[27]
a	chr2:155,050,001-155,070,000	1	[21]
Ccdc73	chr2:104,930,001-104,950,000	1	[39]
Cpd	chr11:76,780,001-76,800,000	1	[22]
Dgki	chr6:37,270,001-37,290,000	1	[41]
En2	chr5:28,160,001-28,180,000	1	[9]
Entpd7	chr19:43,700,001-43,720,000	1	[22]
Ide	chr19:37,300,001-37,320,000	1	[39]
Immp1l	chr2:105,910,001-105,930,000	1	[39]
Oasl2	chr5:114,900,001-114,920,000	1	[21]
Pcdh15	chr10:73,880,001-73,900,000	1	[39]
Vps8	chr16:21,500,001-21,520,000	1	[7]

One particularly striking example of a potential false-positive CIS is *Sfi1* (located within the chr11:3,180,001-3,200,000 window), which has been identified by 12 separate SB screens. Based on our findings, along with the previous identification of this region as underrepresented in the reference genome [28], its recurrent detection as a commonly mutated region in tumors appears to be driven by technical bias rather than by a positive selection pressure for its mutation during neoplastic transformation. Interestingly, *Sfi1* was also identified as a potential false-positive CIS through the analysis of unselected SB insertion sites in mES cells [23]. None of the other potential false-positive CISs discovered in this manner overlapped with our list. Because the experimental conditions we used to characterize background transposition were specifically designed to match those of *in vivo* SB mutagenesis screens, our results are particularly relevant to the functional interpretation of insertion site data from such screens.

## Conclusions

The results that we have described represent significant advancements to the field of cancer gene discovery utilizing insertional mutagenesis models. Our characterization of footprints induced by SB transposon remobilization is the first large-scale analysis of this phenomenon’s prevalence in tumors. We report that remobilization is a relatively rare event in SB-induced tumors and that it is unlikely to result in mutations being missed by traditional screening methods. Through the use of a standard curve experiment, we found that individual insertion sites within heterogeneous tumors can reliably be divided into groups based on a semi-quantitative assignment of clonality. This information will serve to deepen our understanding of the dynamics of mutation acquisition and selection in developing tumors. Finally, we report several genomic regions that are preferentially detected under non-selective conditions *in vivo* by methods currently used to identify SB insertion sites in tumors. By shedding light on this potential source of bias, our results reveal an important factor to consider when interpreting insertion site data. Overall, the methods and data that we have presented will facilitate improved identification of cancer genes by insertional mutagenesis strategies, as well as the enhanced interpretation of data generated using this approach.

## Methods

### Mice

Triple transgenic *CD4*-Cre; *ROSA26-LsL-SBase*; T2/Onc2 mice used for experiments to detect transposon remobilization were generated previously by crossing *CD4-Cre* transgenic mice (Taconic Farms model #4196) to RosaSBase^LsL^;TG6113 double transgenic mice [5]. *ROSA26-CreERT2* mice were purchased from The Jackson Laboratory (Strain B6.129-*Gt(ROSA)26Sor*^*tm1(cre/ERT2)*Tyj^/J; Stock #008463). SB mouse strains carrying the Cre-inducible *ROSA26-LsL-SBase* allele along with one of four distinct transposon concatemers (TG6070, TG6113, TG12740, or TG12775) were generated by the Dupuy laboratory, as previously described [42].

All animal experiments were performed using procedures approved and monitored by the Institutional Animal Care and Use Committee at the University of Iowa. At eight weeks of age, two male and two female mice of each SB strain (TG6070, TG6113, TG12740, and TG12775) were injected intraperitoneally with a single dose of 2 mg tamoxifen (Sigma-Aldrich product # T5648) dissolved in corn oil. Resulting ubiquitous transposon mobilization due to activation of the *ROSA26-LsL-SBase* allele was allowed to proceed for two weeks, at which point mice were euthanized and tissues were collected.

### Detection and validation of transposition footprints in SB-induced tumors

Tumor DNA was isolated, acoustically sheared, end-repaired, and ligated to a blunt adaptor (annealed Linker + and Linker- oligos) as previously described [8]. After blunt adaptor ligation, samples were enzymatically digested with *Hpy*CH4III (New England Biolabs), followed by heat-inactivation at 80**°** for 20 minutes. Unique footprinting adaptor oligos (*Hpy*Linker + and *Hpy*Linker-) were annealed by heating to 95**°** for 5 minutes and cooling slowly to room temperature, leaving a single nucleotide A or T overhang on one end. Annealed adaptors were ligated to digested tumor DNA overnight at 16**°** using T4 DNA ligase (New England Biolabs). Samples were purified using the MinElute 96 UF PCR purification kit (Qiagen) and resuspended in 20 μl of water prior to a two-stage PCR amplification protocol. Primary PCR was conducted using 3 μl DNA as template with a forward primer complementary to the blunt adaptor region (Linker Primer) and a barcoded reverse primer complementary to the *Hpy*CH4III adaptor region (*Hpy*Linker Primer), each included at a final concentration of 200 nM. Platinum Taq DNA polymerase (Life Technologies) was used for amplification with 30 cycles of: 94**°** for 30 sec, 57**°** for 30 sec, and 72**°** for 60 sec. Primary PCR products were diluted 1:50 in water and incubated at room temperature for 30 min. Secondary PCR was conducted using 8 μl of diluted products as template with a nested primer complementary to the blunt adaptor region (Linker-A2 Primer) and the same barcoded reverse primer, each included at a final concentration of 200nM. Cycling conditions were the same as for primary PCR, except that 20 cycles were performed instead of 30. A portion of each reaction was analyzed by electrophoresis on a 1.5% agarose gel to assess library quality. The remaining secondary PCR products were purified using the MinElute 96 UF PCR purification kit (Qiagen). Approximately 25 ng of each sample was pooled and submitted for sequencing on the Illumina Hi-Seq platform. Oligonucleotide sequences used for footprint detection are listed in Additional file 6: Table S4.

Sequences were trimmed and mapped using the IAS pipeline as previously described [4]. The footprint library preparation method will amplify both actual SB-induced footprints as well as the endogenous sequences 5’-TACWGT-3’ and 5’-ACWGTA-3’. A normal genomic DNA sample, prepared and sequenced in the same manner, was used to identify any endogenous sites also amplified by the footprint detection method. Sites present in the normal sample were subtracted from each of the tumor samples. In addition, we assumed that footprint sites would be unique to each sample. Therefore, we also removed any redundant sites present in more than one tumor sample. Finally, we required reads from both the plus- and minus-strands be detected to consider a candidate site to be a *bona fide* footprint. This ensured that the candidate site contained an intact footprint sequence (*i.e.* 5’-TACWGTA-3’).

For validation, ten primer pairs (Additional file 7: Table S5) were designed for each tumor sample to amplify fragments containing a candidate footprint site. PCR products were purified using the MinElute 96 UF PCR Purification Kit (Qiagen), enzymatically digested with *Hpy*CH4III (New England Biolabs), and separated by electrophoresis on 1.5% agarose gels.

### Construction of transposon standard plasmids

Twenty PCR primers were designed to bind genomic DNA sequences adjacent to previously identified transposon insertion sites. Each was used in combination with a transposon-specific primer to amplify fragments containing transposon/genome junctions from tumor DNA samples with Phusion DNA polymerase (New England Biolabs). PCR products were sequenced for verification. Chimeric transposon/genome junctions were cloned into pT2/Onc2 vectors [6] to simulate insertion sites when mixed with tumor DNA samples. Standard plasmid cocktails were constructed via addition of individual plasmids at defined concentrations to mimic insertions found at 100%, 50%, or 12.5% abundance within cells in a given tumor (see Additional file 3: Table S2 for additional information on standard clones).

### LM-PCR library preparation and sequencing

Transposon insertion sites in mouse tissues were sequenced using the Illumina Hi-Seq platform as previously described [8]. Briefly, DNA was isolated from tissues using the GenElute Mammalian Genomic DNA Miniprep Kit (Sigma-Aldrich). Samples were acoustically sheared to an average size of 300 bp using a 96-well E220 sonicator (Covaris) and Illumina library preparation was completed through ligation-mediated PCR (LM-PCR). Sequence reads were mapped and annotated using Bowtie2 software [43].

The false-discovery rate was estimated by generating three independent LM-PCR profiles for six different tumor samples. For each tumor, we treated each single insertion profile as a “test set” of common insertion events found in the remaining two replicate samples (defined as the “true-positive set”). Next, the percentage of false-positive sites (*i.e.* sites present in the test set but absent in the true-positive set) was determined based on this comparison.

### Detection of regions with enriched insertion

The Poisson Regression Insertion Model (PRIM) was used to calculate the expected insertion rate for non-overlapping 20 kilobase windows along the length of each chromosome in the mouse reference genome [17]. The PRIM algorithm generated a statistical model based on the number of TA dinucleotides within each window, the chromosome in which the window resides, and the total number of unique insertions. For each window, the expected number of insertions was calculated and compared to the observed number of insertions to produce a p-value. Bonferroni-correction was then applied to identify windows that showed enrichment for detection of inserted transposons. The processed data sets obtained by mapping using both the IAS and the map-corrected IAS method (IASmc) are provided along with sample information as Additional file 8: Supplemental data.

### Correction method to remove ambiguous mapping events

First, we identified the minimum read length required to unambiguously map sequences surrounding each TA dinucleotide in the mouse reference genome (GRCm38/mm10). To do this, mock sequence reads were generated on both DNA strands starting at a length of 10 bp and increasing in 5 bp increments to a maximum length of 65 bp. Fragments were mapped using Bowtie2 (parameters: −-very-sensitive, −k 10, −-no-hd, and --no-seq). Using these data, the minimum read length required for accurate and unique mapping was determined on both strands for each TA in the reference genome. For this purpose, mapping was characterized as accurate and unique when a mock read mapped to the originating locus with a score >10 and without mapping to any other genomic site having two or more mismatches. We modified our insertion site analysis pipeline such that any experimental sequence read not meeting the minimum required length for accurate and unique mapping was discarded. The remainder of the pipeline functions as previously described [4].

## Electronic supplementary material

Additional file 1: Figure S1-S5: (PDF 3 MB)

Additional file 2: Table S1: Putative SB transposon footprints identified by Illumina sequencing. (XLSX 66 KB)

Additional file 3: Table S2: Characteristics of transposon standard plasmid constructs. (XLSX 53 KB)

Additional file 4: Table S3: Genomic windows with significant enrichment for detected transposon insertions in unselected tissues. (XLSX 102 KB)

Additional file 5: **Supplemental Methods.** Genotyping protocols. (DOCX 110 KB)

Additional file 6: Table S4: Oligonucleotide sequences used for SB footprint sequencing library preparation. (XLSX 26 KB)

Additional file 7: Table S5: Oligonucleotide sequences used for validation of candidate footprints. (XLSX 48 KB)

Additional file 8: **Supplemental data.** Processed data sets obtained by mapping using both the IAS and the map-corrected IAS method (IASmc). (ZIP 5 MB)

## References

[1] Howell VM (2012). Sleeping beauty–a mouse model for all cancers?. Cancer Lett.

[2] Liu G, Aronovich EL, Cui Z, Whitley CB, Hackett PB (2004). Excision of Sleeping Beauty transposons: parameters and applications to gene therapy. J Gene Med.

[3] Plasterk RH, Izsvak Z, Ivics Z (1999). Resident aliens: the Tc1/mariner superfamily of transposable elements. Trends Genet.

[4] Brett BT, Berquam-Vrieze KE, Nannapaneni K, Huang J, Scheetz TE, Dupuy AJ (2011). Novel molecular and computational methods improve the accuracy of insertion site analysis in Sleeping Beauty-induced tumors. PLoS One.

[5] Berquam-Vrieze KE, Nannapaneni K, Brett BT, Holmfeldt L, Ma J, Zagorodna O, Jenkins NA, Copeland NG, Meyerholz DK, Knudson CM, Mullighan CG, Scheetz TE, Dupuy AJ (2011). Cell of origin strongly influences genetic selection in a mouse model of T-ALL. Blood.

[6] Dupuy AJ, Akagi K, Largaespada DA, Copeland NG, Jenkins NA (2005). Mammalian mutagenesis using a highly mobile somatic Sleeping Beauty transposon system. Nature.

[7] Friedel RH, Friedel CC, Bonfert T, Shi R, Rad R, Soriano P (2013). Clonal expansion analysis of transposon insertions by high-throughput sequencing identifies candidate cancer genes in a PiggyBac mutagenesis screen. PLoS One.

[8] Rogers LM, Olivier AK, Meyerholz DK, Dupuy AJ (2013). Adaptive immunity does not strongly suppress spontaneous tumors in a Sleeping Beauty model of cancer. J Immunol.

[9] Koudijs MJ, Klijn C, van der Weyden L, Kool J, ten Hoeve J, Sie D, Prasetyanti PR, Schut E, Kas S, Whipp T, Cuppen E, Wessels L, Adams DJ, Jonkers J (2011). High-throughput semiquantitative analysis of insertional mutations in heterogeneous tumors. Genome Res.

[10] Carlson CM, Dupuy AJ, Fritz S, Roberg-Perez KJ, Fletcher CF, Largaespada DA (2003). Transposon mutagenesis of the mouse germline. Genetics.

[11] Liang Q, Kong J, Stalker J, Bradley A (2009). Chromosomal mobilization and reintegration of Sleeping Beauty and PiggyBac transposons. Genesis.

[12] Woodard LE, Li X, Malani N, Kaja A, Hice RH, Atkinson PW, Bushman FD, Craig NL, Wilson MH (2012). Comparative analysis of the recently discovered hAT transposon TcBuster in human cells. PLoS One.

[13] Yant SR, Wu X, Huang Y, Garrison B, Burgess SM, Kay MA (2005). High-resolution genome-wide mapping of transposon integration in mammals. Mol Cell Biol.

[14] Ammar I, Gogol-Doring A, Miskey C, Chen W, Cathomen T, Izsvak Z, Ivics Z (2012). Retargeting transposon insertions by the adeno-associated virus Rep protein. Nucleic Acids Res.

[15] Vigdal TJ, Kaufman CD, Izsvak Z, Voytas DF, Ivics Z (2002). Common physical properties of DNA affecting target site selection of sleeping beauty and other Tc1/mariner transposable elements. J Mol Biol.

[16] Ventura A, Kirsch DG, McLaughlin ME, Tuveson DA, Grimm J, Lintault L, Newman J, Reczek EE, Weissleder R, Jacks T (2007). Restoration of p53 function leads to tumour regression in vivo. Nature.

[17] Bergemann TL, Starr TK, Yu H, Steinbach M, Erdmann J, Chen Y, Cormier RT, Largaespada DA, Silverstein KA (2012). New methods for finding common insertion sites and co-occurring common insertion sites in transposon- and virus-based genetic screens. Nucleic Acids Res.

[18] Collier LS, Adams DJ, Hackett CS, Bendzick LE, Akagi K, Davies MN, Diers MD, Rodriguez FJ, Bender AM, Tieu C, Matise I, Dupuy AJ, Copeland NG, Jenkins NA, Hodgson JG, Weiss WA, Jenkins RB, Largaespada DA (2009). Whole-body sleeping beauty mutagenesis can cause penetrant leukemia/lymphoma and rare high-grade glioma without associated embryonic lethality. Cancer Res.

[19] Keng VW, Sia D, Sarver AL, Tschida BR, Fan D, Alsinet C, Sole M, Lee WL, Kuka TP, Moriarity BS, Villanueva A, Dupuy AJ, Riordan JD, Bell JB, Silverstein KA, Llovet JM, Largaespada DA (2013). Sex bias occurrence of hepatocellular carcinoma in Poly7 molecular subclass is associated with EGFR. Hepatology.

[20] Mann KM, Ward JM, Yew CC, Kovochich A, Dawson DW, Black MA, Brett BT, Sheetz TE, Dupuy AJ, Genome I, Chang DK, Biankin AV, Waddell N, Kassahn KS, Grimmond SM, Rust AG, Adams DJ, Jenkins NA, Copeland NG, Australian Pancreatic Cancer (2012). Sleeping Beauty mutagenesis reveals cooperating mutations and pathways in pancreatic adenocarcinoma. Proc Natl Acad Sci U S A.

[21] March HN, Rust AG, Wright NA, ten Hoeve J, de Ridder J, Eldridge M, van der Weyden L, Berns A, Gadiot J, Uren A, Kemp R, Arends MJ, Wessels LF, Winton DJ, Adams DJ (2011). Insertional mutagenesis identifies multiple networks of cooperating genes driving intestinal tumorigenesis. Nat Genet.

[22] Perez-Mancera PA, Rust AG, van der Weyden L, Kristiansen G, Li A, Sarver AL, Silverstein KA, Grutzmann R, Aust D, Rummele P, Knosel T, Herd C, Stemple DL, Kettleborough R, Brosnan JA, Li A, Morgan R, Knight S, Yu J, Stegeman S, Collier LS, ten Hoeve JJ, de Ridder J, Klein AP, Goggins M, Hruban RH, Chang DK, Biankin AV, Grimmond SM, Australian Pancreatic Cancer Genome Initiative (2012). The deubiquitinase USP9X suppresses pancreatic ductal adenocarcinoma. Nature.

[23] de Jong J, Akhtar W, Badhai J, Rust AG, Rad R, Hilkens J, Berns A, van Lohuizen M, Wessels LF, de Ridder J (2014). Chromatin landscapes of retroviral and transposon integration profiles. PLoS Genet.

[24] Mattison J, Kool J, Uren AG, de Ridder J, Wessels L, Jonkers J, Bignell GR, Butler A, Rust AG, Brosch M, Wilson CH, van der Weyden L, Largaespada DA, Stratton MR, Futreal PA, van Lohuizen M, Berns A, Collier LS, Hubbard T, Adams DJ (2010). Novel candidate cancer genes identified by a large-scale cross-species comparative oncogenomics approach. Cancer Res.

[25] Starr TK, Allaei R, Silverstein KA, Staggs RA, Sarver AL, Bergemann TL, Gupta M, O'Sullivan MG, Matise I, Dupuy AJ, Collier LS, Powers S, Oberg AL, Asmann YW, Thibodeau SN, Tessarollo L, Copeland NG, Jenkins NA, Cormier RT, Largaespada DA (2009). A transposon-based genetic screen in mice identifies genes altered in colorectal cancer. Science.

[26] Starr TK, Scott PM, Marsh BM, Zhao L, Than BL, O'Sullivan MG, Sarver AL, Dupuy AJ, Largaespada DA, Cormier RT (2011). A Sleeping Beauty transposon-mediated screen identifies murine susceptibility genes for adenomatous polyposis coli (Apc)-dependent intestinal tumorigenesis. Proc Natl Acad Sci U S A.

[27] Rahrmann EP, Watson AL, Keng VW, Choi K, Moriarity BS, Beckmann DA, Wolf NK, Sarver A, Collins MH, Moertel CL, Wallace MR, Gel B, Serra E, Ratner N, Largaespada DA (2013). Forward genetic screen for malignant peripheral nerve sheath tumor formation identifies new genes and pathways driving tumorigenesis. Nat Genet.

[28] Quinlan AR, Clark RA, Sokolova S, Leibowitz ML, Zhang Y, Hurles ME, Mell JC, Hall IM (2010). Genome-wide mapping and assembly of structural variant breakpoints in the mouse genome. Genome Res.

[29] Abbot KL, Nyre ET, Abrahante J, Ho YY, Isaksson Vogel R, Starr TK (2014). The Candidate Cancer Gene Database: a database of cancer driver genes from forward genetic screens in mice. Nucleic Acids Res.

[30] Bender AM, Collier LS, Rodriguez FJ, Tieu C, Larson JD, Halder C, Mahlum E, Kollmeyer TM, Akagi K, Sarkar G, Largaespada DA, Jenkins RB (2010). Sleeping beauty-mediated somatic mutagenesis implicates CSF1 in the formation of high-grade astrocytomas. Cancer Res.

[31] Keng VW, Villanueva A, Chiang DY, Dupuy AJ, Ryan BJ, Matise I, Silverstein KA, Sarver A, Starr TK, Akagi K, Tessarollo L, Collier LS, Powers S, Lowe SW, Jenkins NA, Copeland NG, Llovet JM, Largaespada DA (2009). A conditional transposon-based insertional mutagenesis screen for genes associated with mouse hepatocellular carcinoma. Nat Biotechnol.

[32] Lastowska M, Al-Afghani H, Al-Balool HH, Sheth H, Mercer E, Coxhead JM, Redfern CP, Peters H, Burt AD, Santibanez-Koref M, Bacon CM, Chesler L, Rust AG, Adams DJ, Williamson D, Clifford SC, Jackson MS (2013). Identification of a neuronal transcription factor network involved in medulloblastoma development. Acta Neuropathol Commun.

[33] O'Donnell KA, Keng VW, York B, Reineke EL, Seo D, Fan D, Silverstein KA, Schrum CT, Xie WR, Mularoni L, Wheelan SJ, Torbenson MS, O'Malley BW, Largaespada DA, Boeke JD (2012). A Sleeping Beauty mutagenesis screen reveals a tumor suppressor role for Ncoa2/Src-2 in liver cancer. Proc Natl Acad Sci U S A.

[34] Quintana RM, Dupuy AJ, Bravo A, Casanova ML, Alameda JP, Page A, Sanchez-Viera M, Ramirez A, Navarro M (2013). A transposon-based analysis of gene mutations related to skin cancer development. J Invest Dermatol.

[35] van der Weyden L, Giotopoulos G, Rust AG, Matheson LS, van Delft FW, Kong J, Corcoran AE, Greaves MF, Mullighan CG, Huntly BJ, Adams DJ (2011). Modeling the evolution of ETV6-RUNX1-induced B-cell precursor acute lymphoblastic leukemia in mice. Blood.

[36] van der Weyden L, Rust AG, McIntyre RE, Robles-Espinoza CD, del Castillo V-HM, Strogantsev R, Ferguson-Smith AC, McCarthy S, Keane TM, Arends MJ, Adams DJ (2013). Jdp2 downregulates Trp53 transcription to promote leukaemogenesis in the context of Trp53 heterozygosity. Oncogene.

[37] Wu X, Northcott PA, Dubuc A, Dupuy AJ, Shih DJ, Witt H, Croul S, Bouffet E, Fults DW, Eberhart CG, Garzia L, Van Meter T, Zagzag D, Jabado N, Schwartzentruber J, Majewski J, Scheetz TE, Pfister SM, Korshunov A, Li XN, Scherer SW, Cho YJ, Akagi K, MacDonald TJ, Koster J, McCabe MG, Sarver AL, Collins VP, Weiss WA, Largaespada DA, Collier LS, Taylor MD (2012). Clonal selection drives genetic divergence of metastatic medulloblastoma. Nature.

[38] Koso H, Takeda H, Yew CC, Ward JM, Nariai N, Ueno K, Nagasaki M, Watanabe S, Rust AG, Adams DJ, Copeland NG, Jenkins NA (2012). Transposon mutagenesis identifies genes that transform neural stem cells into glioma-initiating cells. Proc Natl Acad Sci U S A.

[39] Bard-Chapeau EA, Nguyen AT, Rust AG, Sayadi A, Lee P, Chua BQ, New LS, de Jong J, Ward JM, Chin CK, Chew V, Toh HC, Abastado JP, Benoukraf T, Soong R, Bard FA, Dupuy AJ, Johnson RL, Radda GK, Chan EC, Wessels LF, Adams DJ, Jenkins NA, Copeland NG (2014). Transposon mutagenesis identifies genes driving hepatocellular carcinoma in a chronic hepatitis B mouse model. Nat Genet.

[40] Tang JZ, Carmichael CL, Shi W, Metcalf D, Ng AP, Hyland CD, Jenkins NA, Copeland NG, Howell VM, Zhao ZJ, Smyth GK, Kile BT, Alexander WS (2013). Transposon mutagenesis reveals cooperation of ETS family transcription factors with signaling pathways in erythro-megakaryocytic leukemia. Proc Natl Acad Sci U S A.

[41] Genovesi LA, Ng CG, Davis MJ, Remke M, Taylor MD, Adams DJ, Rust AG, Ward JM, Ban KH, Jenkins NA, Copeland NG, Wainwright BJ (2013). Sleeping Beauty mutagenesis in a mouse medulloblastoma model defines networks that discriminate between human molecular subgroups. Proc Natl Acad Sci U S A.

[42] Dupuy AJ, Rogers LM, Kim J, Nannapaneni K, Starr TK, Liu P, Largaespada DA, Scheetz TE, Jenkins NA, Copeland NG (2009). A modified sleeping beauty transposon system that can be used to model a wide variety of human cancers in mice. Cancer Res.

[43] Langmead B, Salzberg SL (2012). Fast gapped-read alignment with Bowtie 2. Nat Methods.

