# Supplementary material for: Sequencing methods and datasets to improve functional interpretation of sleeping beauty mutagenesis screens
Source: BMC Genomics. 2014 Dec 19;15(1):1150. doi: 10.1186/1471-2164-15-1150 (PMC4378557; doi:10.1186/1471-2164-15-1150)
Supplement: Supplementary file 1 — Additional file 1: Figure S1-S5: (PDF 3 MB) [file 12864_2014_6920_MOESM1_ESM.pdf]

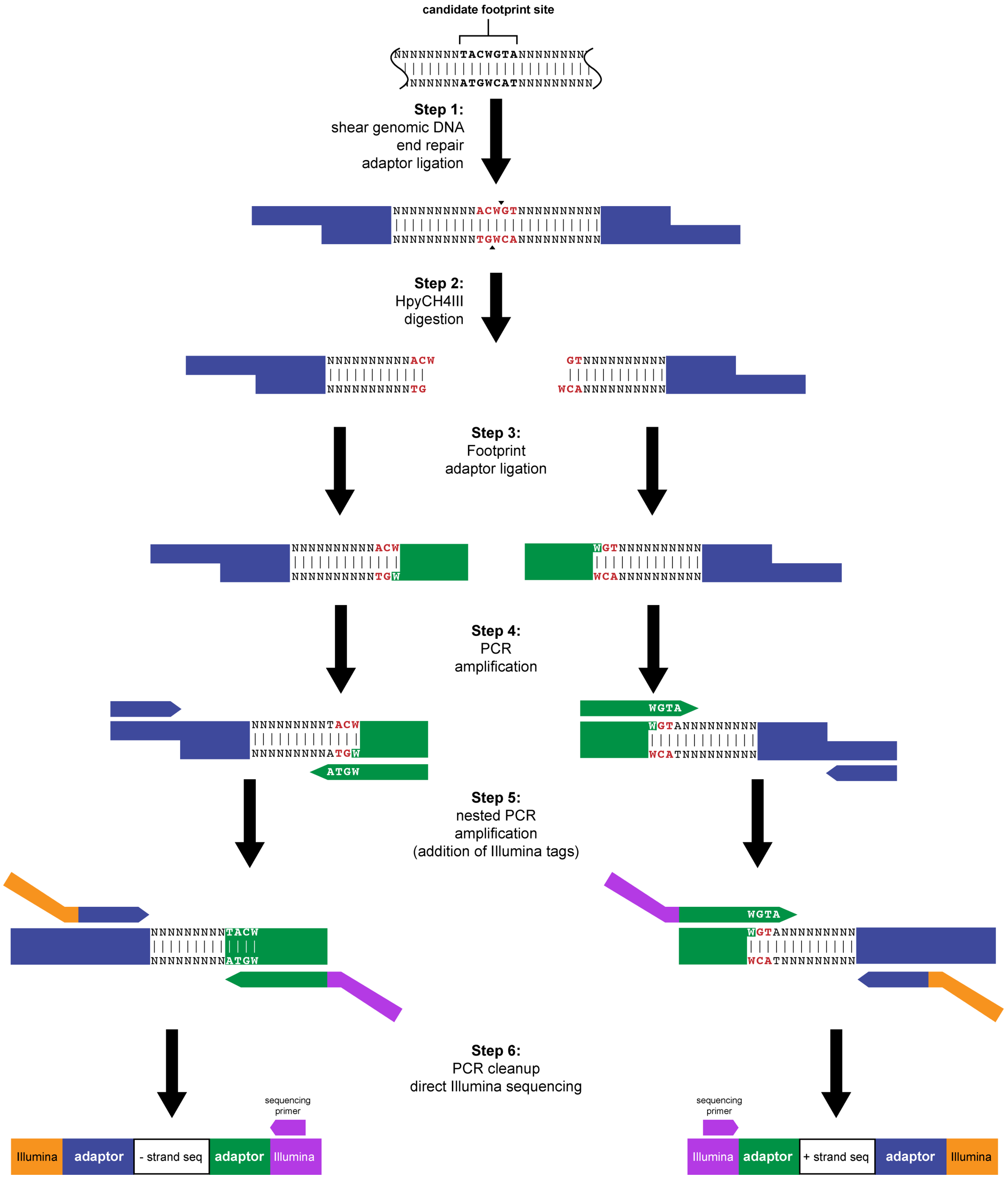

Figure S1. Overview of method to isolate candidate footprint sites from SB-induced tumors. A canonical SB footprint converts a TA dinucleotide to the sequence TACWGTA, where the "W" position is either an adenine or thymine residue. Thus the footprint sequence contains a recognition site for the HpyCh4III restriction enzyme. The HpyCH4III site was used in the genomic library preparation to add a second adaptor tag containing a specific primer sequence. This strategy reduced the complexity of the library by selectively amplifying genomic HpyCH4III sites, of which SB footprints will be a subset. This strategy was then able to identify candidate footprint sites by comparing the sites identified in a matched normal control sample to that of SB-induced tumors. (See Methods section for primer sequences and protocol).

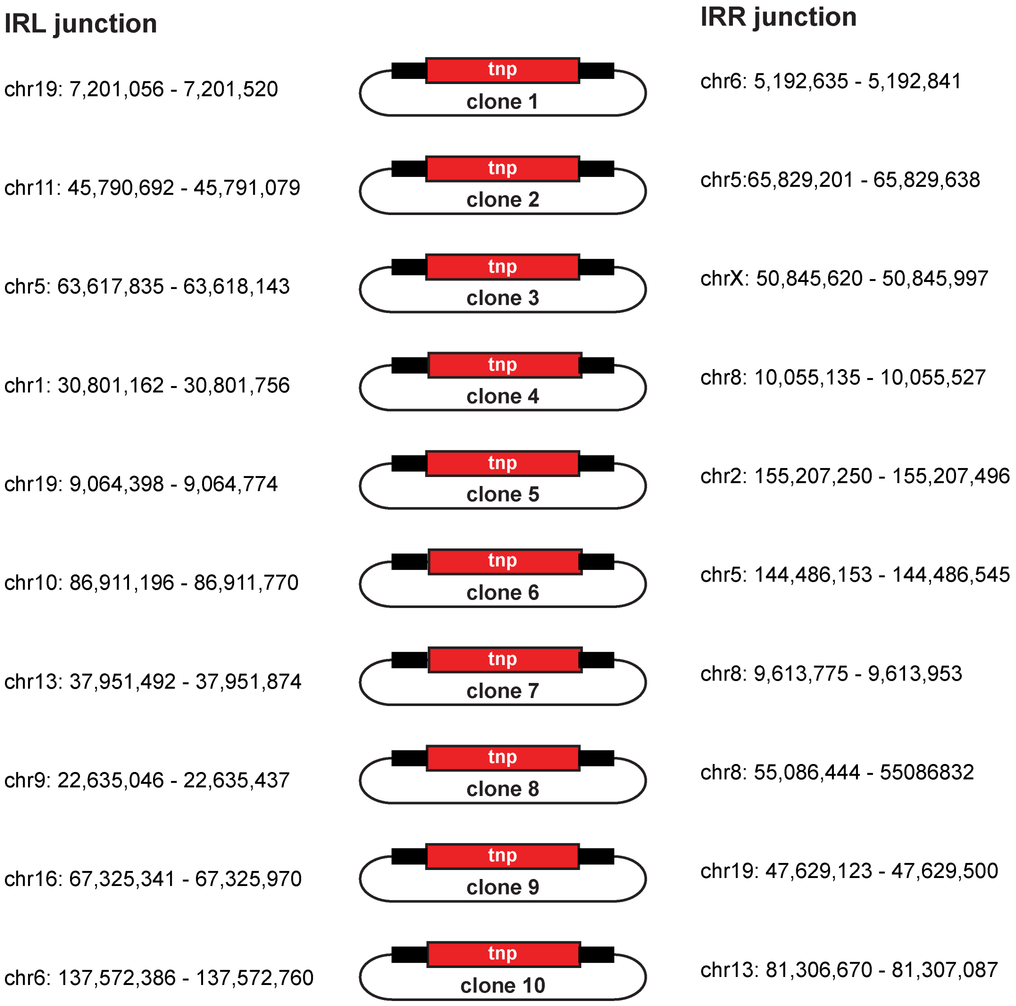

Figure S2. Overview of standard clone collection. A plasmid containing a single copy of the T2/Onc2 transposon (tnp) was modified to generate specific transposon/genome junctions previously identified in DNA from mouse tumors (listed coordinates refer to the GRCm38/mm10 reference genome). These clones were mixed at varying ratios and spiked into tumor DNA (see Methods).

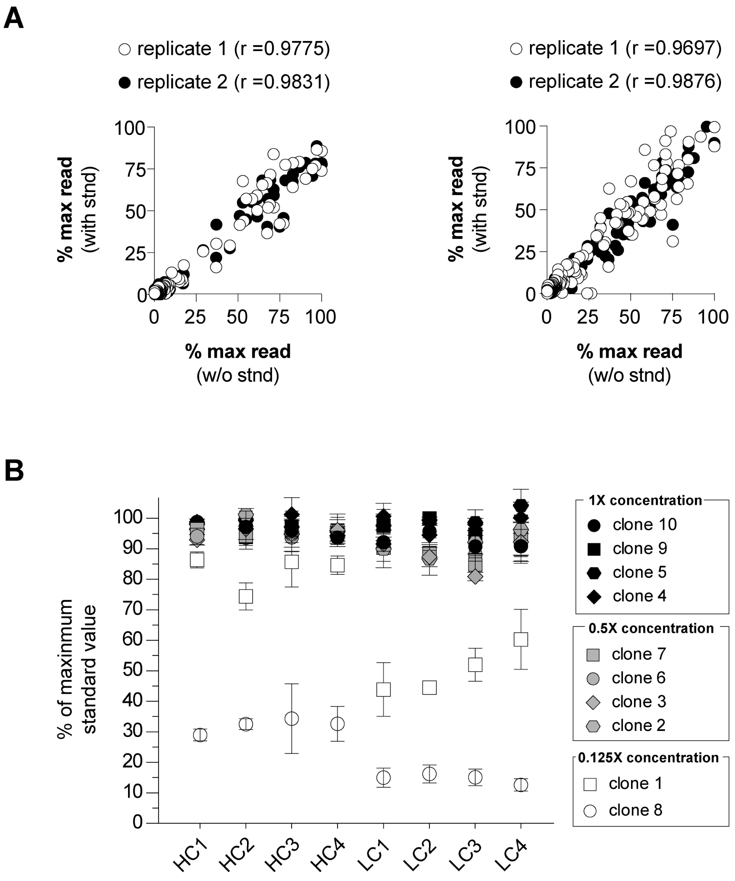

Figure S3. Performance of standards in ligation-mediated PCR. (A) The standards were spiked into two SB-induced tumors, and the mixed samples were processed twice independently. The SB-induced transposon insertions were examined to determine if the presence of the standards significantly altered the read distribution. The results from both samples show that the standards do not perturb the amplification of transposon junctions. **(B)** A prior publication by Koudijs et al. suggested that the number of break points (i.e. ligation points) generated by acoustic shearing of the tumor DNA for each insertion site provides some quantitative information about the abundance of each insertion in the sample. Our results indicate that the number of break points does not perform as well as the normalized read number in measuring the abundance of each standard clone. This was true of tumor samples that were derived from mice carrying high-copy (HC1-4) and low-copy (LC1-4) transposon arrays.

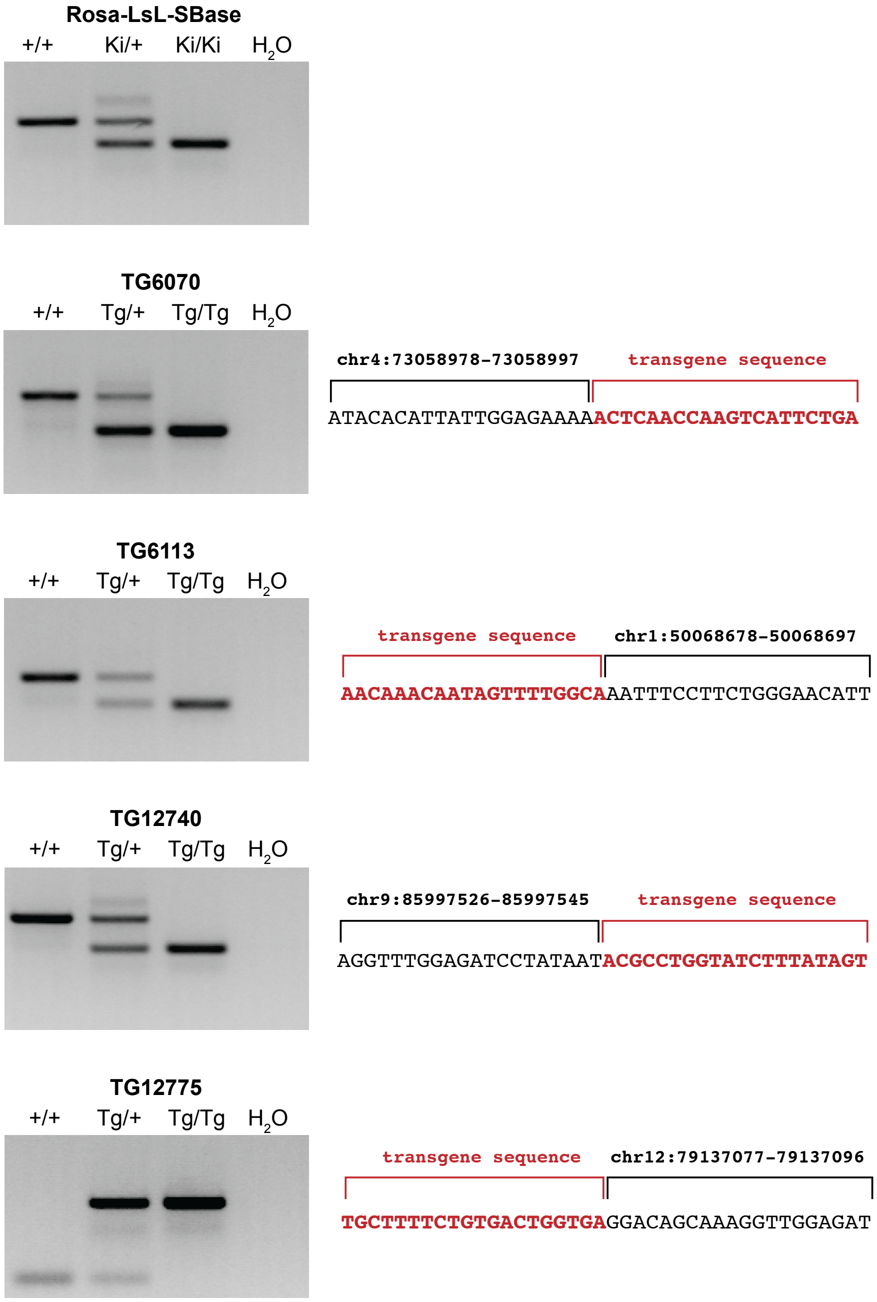

Figure S4. Genotyping SB mouse strains. Amplified products resulting from genotyping PCRs were separated by electrophoresis on 1.5% agarose gels. Products amplified from the genomic DNA of wild-type mice, heterozygotes, and homozygotes for the transgene of interest are depicted, along with a control reaction lacking DNA template. Sequences adjacent to the gel image for each of the four transposon alleles (TG6070, TG6113, TG12740, and TG12775) show the junction between the transposon transgene concatemer and genomic DNA for that strain. Transposon sequence is highlighted in red, and genomic coordinates on genome assembly GRCm38/mm10 are listed for the bases shown.

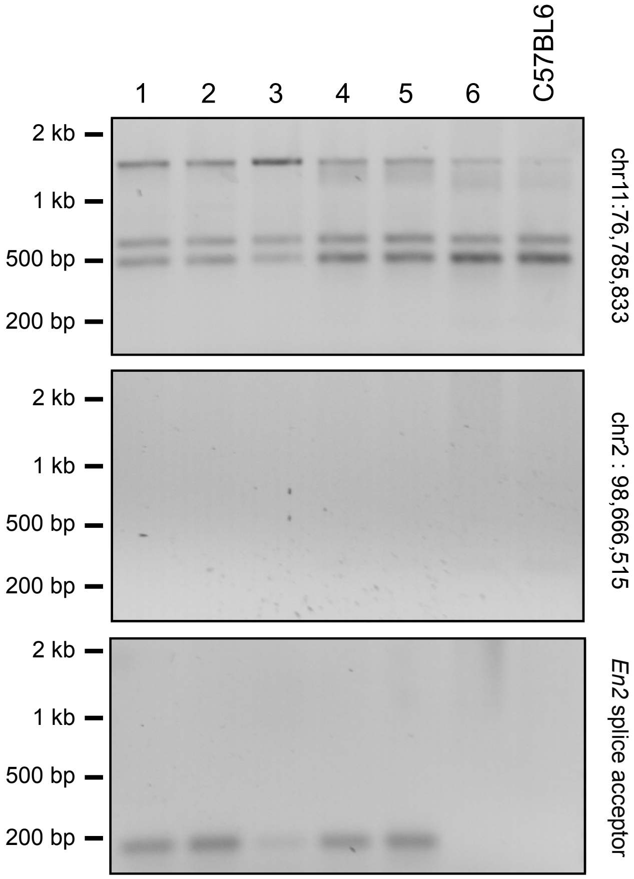

Locus	primer sequences	expected product size(s):
chr11: 76,785,833	5'- CCATGGCAACACTGACTGAC -3'<br>5'- TGTATGTAAACTTCCGACTTCAACTG -3'	214 bp
chr2: 98,666,515	5'- AAGCCTTTCCCCAATCTTTC -3'<br>5'- TGTATGTAAACTTCCGACTTCAACTG -3'	353 bp
En2 splice acceptor	5'- TGGGATCCCCTAGTTTGTGA -3'<br>5'- TGTATGTAAACTTCCGACTTCAACTG -3'	115 - 160 bp

Figure S5. PCR verification of background insertion events produced by mapping errors. Two genomic TA sites were identified as likely sites of false-mapping due to the Bowtie2 short read sequence aligner used in our pipeline. Primers were designed to amplify transposon/genome junctions from six DNA samples that appeared to harbor insertions at the indicated position. Genomic DNA from a wild type C57BL6 mouse was used as a negative control. As expected, PCR amplification produced either background products (top) or no detectable products (middle) for the suspected background sites. By contrast, PCR products could be readily amplified from the *En2* splice acceptor region in five of the six samples. This sequence is present in all transposons, and prior work has shown that transposition within the original transgene often results in insertions in this region. Such events are interpreted to be local hopping events.
