# Supplementary material for: Sequencing methods and datasets to improve functional interpretation of sleeping beauty mutagenesis screens
Source: BMC Genomics. 2014 Dec 19;15(1):1150. doi: 10.1186/1471-2164-15-1150 (PMC4378557; doi:10.1186/1471-2164-15-1150)
Supplement: Supplementary file 5 — Additional file 5: Supplemental Methods. Genotyping protocols. (DOCX 110 KB) [file 12864_2014_6920_MOESM5_ESM.docx]

**Genotyping protocols**

*Rosa26-LsL-SBase*

Rosa26L:  5’ – CACTTGCTCTCCCAAAGTCGCT – 3’
Rosa26R:  5’ – GGCGGATCACAAGCAATAATAACC – 3’
Rosa26SBLsLR:  5’ – GGGGTGGTGATATAAACTTGAGGCT – 3’

For the PCR, a final concentration of 80nM was used for each of the three primers.  The wild-type allele yields a product of 444bp resulting from amplification using the Rosa26L and Rosa26R primers.  The SB LsL knock-in allele yields a product of 342bp, resulting from amplification using the Rosa26L and Rosa26SBLsLR primers.  Both products are amplified for heterozygous animals.

*TG6070*

6070wt L: 5’ – GCAAACAGAACCCTCCAAAA – 3’

6070wt R: 5’ – TTGGTGATGAAGTTGCAGGA – 3’

6070onc2 R: 5’ – CGCCGCATACACTATTCTCA – 3’

For the PCR, a final concentration of 200nM for each of the wt primers and 120nM for the Onc2 primer was used.  The wild-type allele yields a product of 433 bp resulting from amplification using the 6070wt L and 6070wt R primers, and the transgenic T2/Onc2 allele yields a product of 281 bp resulting from amplification using the 6070wt L and 6070onc2 R primers.  Both bands show up for heterozygous animals.

*TG6113*

6113wt L:  5' – TGGTTGCTGAGGGTTAAACA – 3'
6113wt R:  5' – AGCATCTCCTTGGGCCTACT – 3'
6113onc2 L:  5' – CAAACAATAGTTTTGGCAAATTTC – 3'

For the PCR, a final concentration of 80nM for each of the wt primers and 320nM for the Onc2 primer was used.  The wild-type allele yields a product of 338 bp resulting from amplification using the 6113wt L and 6113wt R primers, and the transgenic T2/Onc2 allele yields a product of 238 bp resulting from amplification using the 6113onc2 L and 6113wt R primers.  Both bands show up for heterozygous animals.

*TG12740*

12740wt L:  5' – ATGAGATGGGAGCAGTGGAG – 3'
12740wt R:  5' – GCAGCTTTTTCTGGACACCT – 3'
12740onc3 R:  5' – CTGGTTTATTGCTGGCGTTT – 3'

For the PCR, a final concentration of 100nM for each of the wt primers and 200nM for the Onc3 primer was used.  The wild-type allele yields a product of 457 bp resulting from amplification using the 12740wt L and 12740wt R primers, and the transgenic T2/Onc3 allele yields a product of 315 bp resulting from amplification using the 12740wt L and 12740onc3 R primers.  Both bands show up for heterozygous animals.

*TG12775*

12775wt L: 5' - GTGATGGGAGATGGAAATGG - 3'
12775wt R: 5' - TGCTTACCCATCTCCAACCT - 3'
12775onc3 L: 5' - AACTTTATCCGCCTCCATCC - 3'

For the PCR, a final concentration of 100nM for each of the wt primers and 200nM for the Onc3 primer was used.  The wild-type allele yields a product of 105bp resulting from amplification using the 12775wt L and 12775wt R primers, and the transgenic T2/Onc3 allele yields a product of 362bp resulting from amplification using the 12775onc3 L and 12775wt R primers.  Both bands show up for heterozygous animals.

*Cycling conditions*

For all reactions, cycling was conducted as follows:

94˚C    2:00

94˚C    0:30
55˚C    0:30
72˚C    0:30
        Repeat 35X

72˚C    1:00
